# Supplementary material for: Reactive Comb Polymer Compatibilized Immiscible PVDF/PLLA Blends: Effects of the Main Chain Structure of Compatibilizer
Source: Polymers (Basel). 2020 Mar 2;12(3):526. doi: 10.3390/polym12030526 (PMC7182944; doi:10.3390/polym12030526)
Supplement: Supplementary file 1 [file polymers-12-00526-s001.pdf]

polymers

MDPI

*Supporting Information*

# Reactive Comb Polymer Compatibilized Immiscible PVDF/PLLA Blends: Effects of the Main Chain Structure of Compatibilizer

**Xin Yang [1], Jinxing Song [2], Hengti Wang [1], Qingqing Lin [1], Xianhua Jin [2], Xin Yang [2] and Yongjin Li [1,*]**

[1] College of Materials, Chemistry and Chemical Engineering, Hangzhou Normal University, No. 2318 Yuhangtang Rd., Hangzhou 311121, China; yangxin@stu.hznu.edu.cn (X.Y.); hengti-wang@hznu.edu.cn (H.W.); lqq0770709@163.com (Q.L.)

[2] Transfar Zhilian Co. Ltd., Hangzhou 311215, P.R. China; songjinxing@126.com (J.S.); gfjxh@163.com (X.J.); 11002@etransfar.com (X.Y.)

* Correspondence: yongjin-li@hznu.edu.cn; Tel.: +86 57128867026

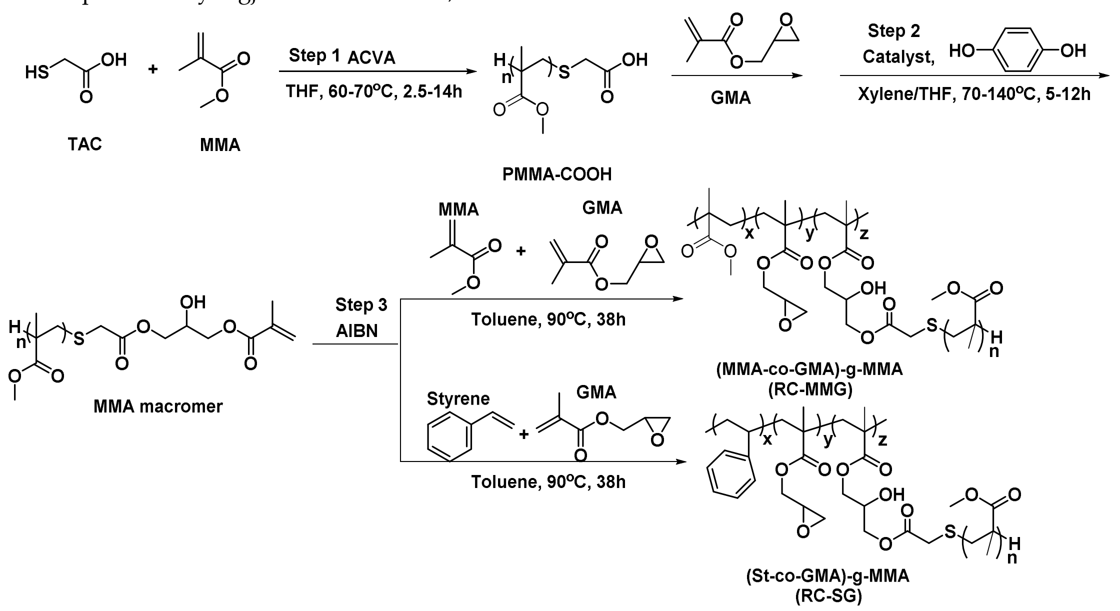


**Scheme S1.** The specific synthesis routes of RC–SG and RC–MMG.

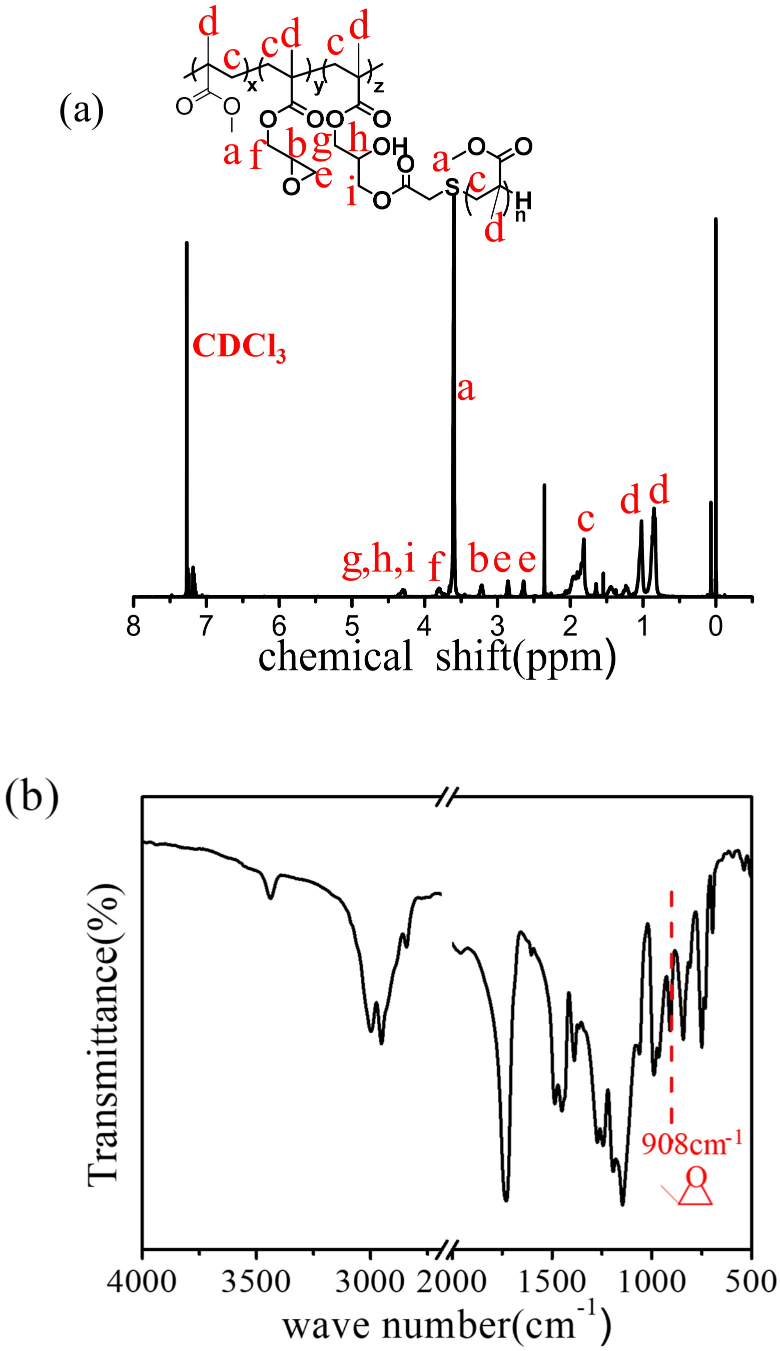


**Figure S1.** $^1$H-NMR and IR spectrum of RC–MMG.

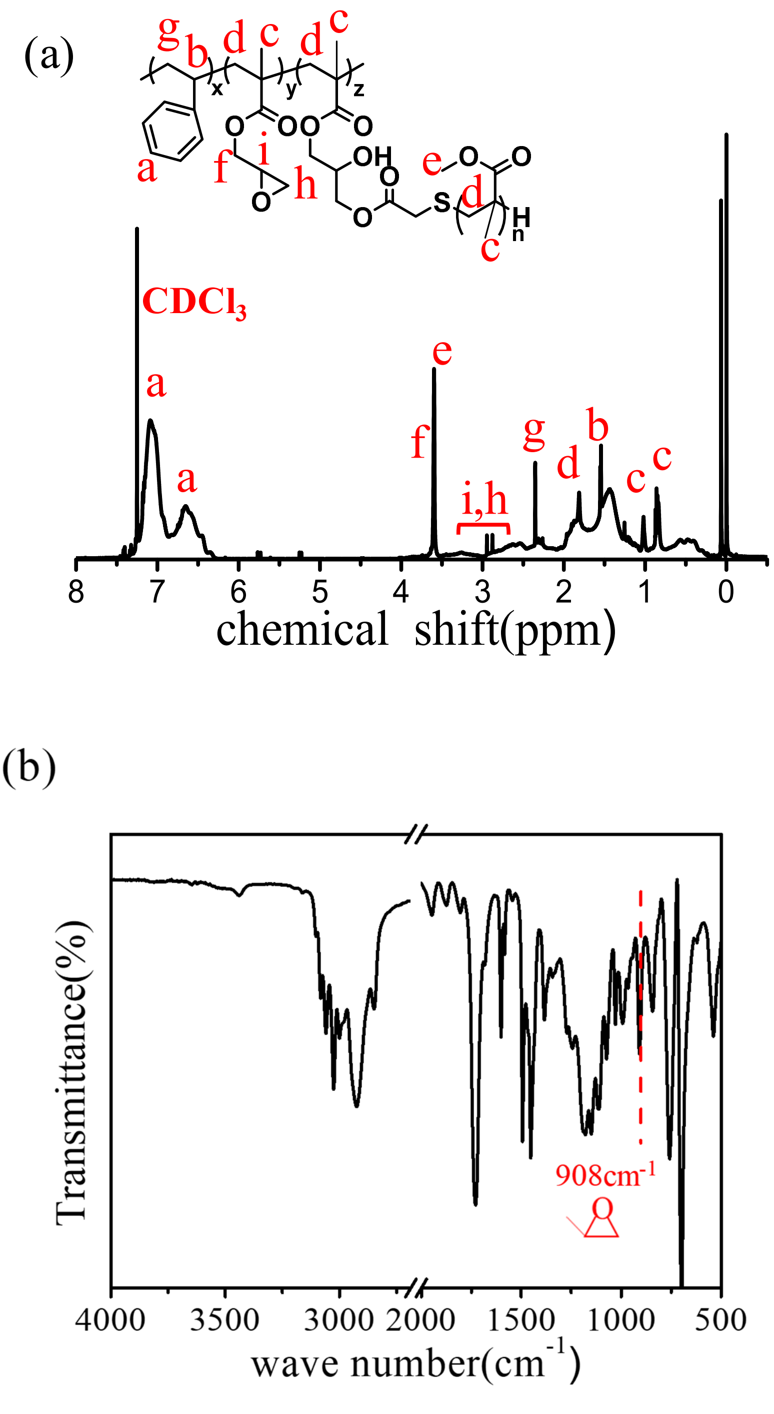


**Figure S2.** $^{1}$H-NMR and IR spectrum of RC–SG.

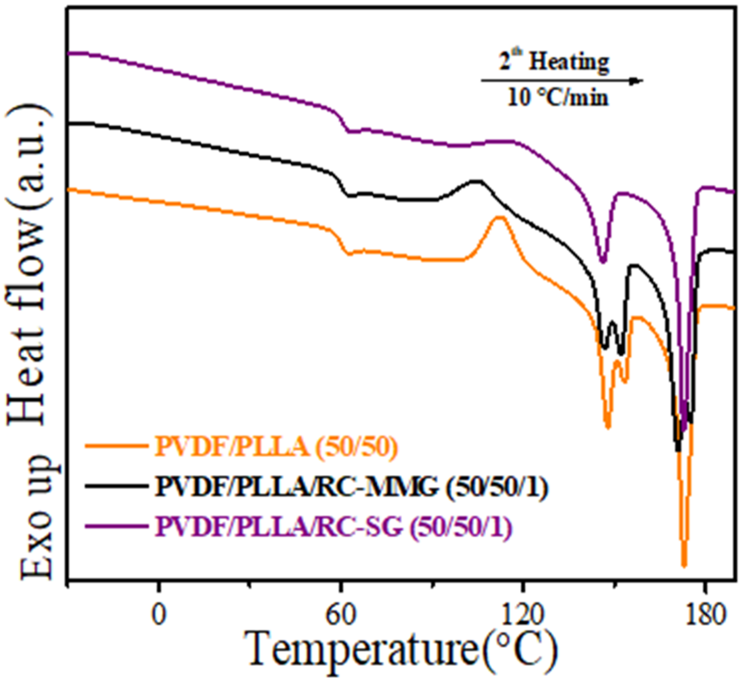


**Figure S3.** DSC curves of PVDF/PLLA (50/50) blend without and with various compatibilizers.

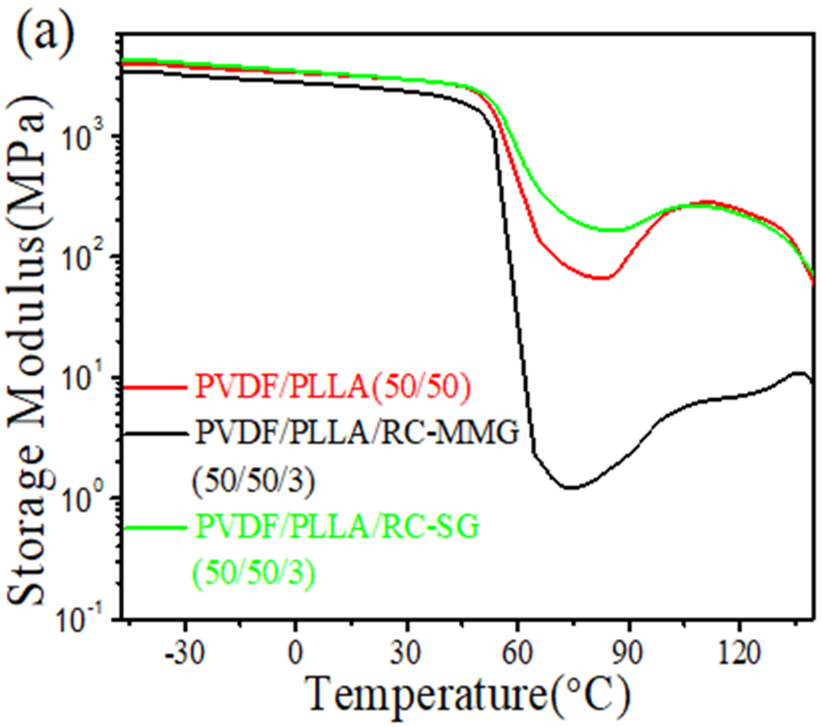

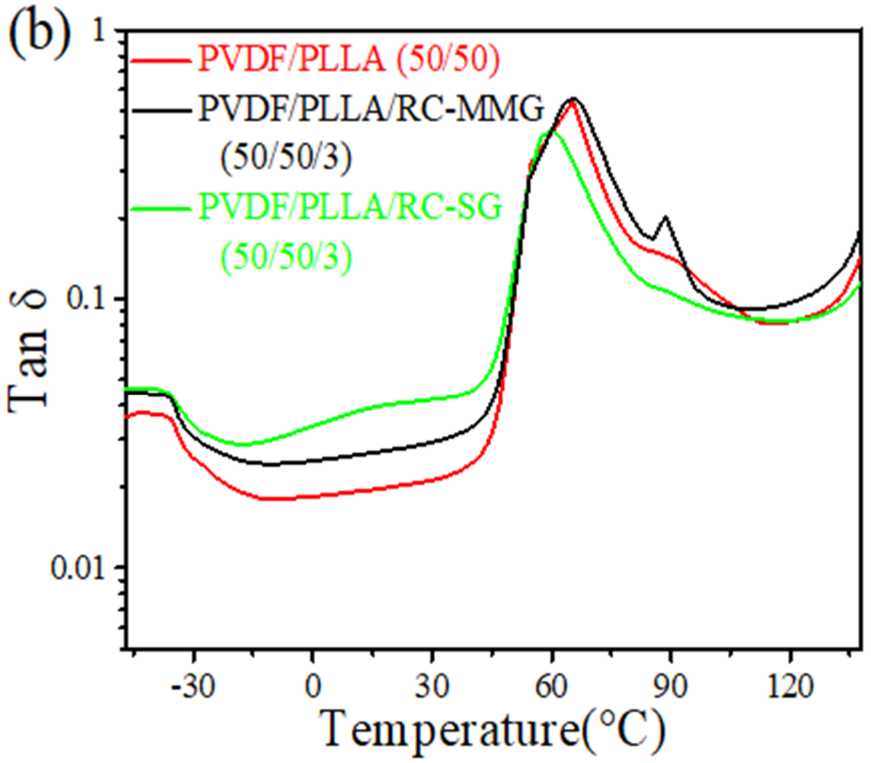


**Figure S4.** DMA curves of PVDF/PLLA (50/50) blend without and with various compatibilizers.

**Table S1.** Mechanical properties for PVDF/PLLA blends with and without compatibilizers.

| Sample Code | Breaking Strength (Mpa) | Modulus (Mpa) | Elongation at Break (%) | Impact Strength (kJ/m²) |
|---|---|---|---|---|
| PVDF/PLLA 50/50 | 40.8 ± 0.9 | 1257.4 ± 60.4 | 3.6 ± 0.3 | 12.5 ± 1.5 |
| PVDF/PLLA/RC-MMG 50/50/0.5 | 35.9 ± 1.3 | 1225.6 ± 85.3 | 109.7 ± 43.2 | 20.8 ± 5.0 |
| PVDF/PLLA/RC-SG 50/50/0.5 | 40.2 ± 1.5 | 1255.1 ± 16.8 | 159.0 ± 32.3 | 23.0 ± 10.0 |
| PVDF/PLLA/RC-MMG 50/50/1 | 37.6 ± 2.0 | 1226.1 ± 78.9 | 135.5 ± 20.1 | 29.5 ± 12.5 |
| PVDF/PLLA/RC-SG 50/50/1 | 46.1 ± 2.8 | 1248.7 ± 48.8 | 252.2 ± 70.1 | 36.3 ± 7.5 |
| PVDF/PLLA/RC-MMG 50/50/3 | 46.2 ± 3.8 | 1331.6 ± 49.5 | 340.4 ± 39.8 | 33.0 ± 10.0 |
| PVDF/PLLA/RC-SG 50/50/3 | 59.1 ± 3.3 | 1352.5 ± 30.0 | 408.4 ± 37.4 | 41.5 ± 2.5 |
